# Extremely high conductivity observed in the triple point topological metal MoP

**DOI:** 10.1038/s41467-019-10126-y

**Published:** 2019-06-06

**Authors:** Nitesh Kumar, Yan Sun, Michael Nicklas, Sarah J. Watzman, Olga Young, Inge Leermakers, Jacob Hornung, Johannes Klotz, Johannes Gooth, Kaustuv Manna, Vicky Süß, Satya N. Guin, Tobias Förster, Marcus Schmidt, Lukas Muechler, Binghai Yan, Peter Werner, Walter Schnelle, Uli Zeitler, Jochen Wosnitza, Stuart S. P. Parkin, Claudia Felser, Chandra Shekhar

**Affiliations:** 1 Max Planck Institute for Chemical Physics of Solids, 01187 Dresden, Germany; 2 Department of Mechanical and Aerospace Engineering, The Ohio State University, Columbus, Ohio 43210 USA; 3 High Field Magnet Laboratory (HFML-EMFL) and Institute for Molecules & Materials, Radboud University, Toernooiveld 7, 6525 ED Nijmegen, The Netherlands; 4 Dresden High Magnetic Field Laboratory (HLD-EMFL), Helmholtz-Zentrum Dresden-Rossendorf, 01328 Dresden, Germany; 5 Institute for Solid-State and Material Physics, Technical University Dresden, 01062 Dresden, Germany; 6 Department of Chemistry, Princeton University, Princeton, NJ 08544 USA; 7 Department of Condensed Matter Physics, Weizmann Institute of Science, 7610001 Rehovot, Israel; 8 Max Planck Institute of Microstructure Physics, 06120 Halle, Germany; 9 Present Address: Department of Mechanical and Material Engineering, University of Cincinnati, Cincinnati, 45219 USA

**Keywords:** Electronic properties and materials, Topological insulators

## Abstract

Weyl and Dirac fermions have created much attention in condensed matter physics and materials science. Recently, several additional distinct types of fermions have been predicted. Here, we report ultra-high electrical conductivity in MoP at low temperature, which has recently been established as a triple point fermion material. We show that the electrical resistivity is 6 nΩ cm at 2 K with a large mean free path of 11 microns. de Haas-van Alphen oscillations reveal spin splitting of the Fermi surfaces. In contrast to noble metals with similar conductivity and number of carriers, the magnetoresistance in MoP does not saturate up to 9 T at 2 K. Interestingly, the momentum relaxing time of the electrons is found to be more than 15 times larger than the quantum coherence time. This difference between the scattering scales shows that momentum conserving scattering dominates in MoP at low temperatures.

## Abstract (web summary)

Although novel topological quasiparticles have recently been evidenced, their electrical transport properties remain elusive. Here, the authors report ultra-low resistivity down to 6 nΩcm at 2 K in MoP with a large mean free path, which hints on the exotic properties of triple point fermions.

## Introduction

Materials are conventionally divided into metals, semiconductors, and insulators. Recently, through the lens of topology, materials can be reclassified as either topologically trivial or topologically non-trivial. Topologically non-trivial semimetals and metals exhibit novel, low-energy fermionic excitations of which the most extensively studied are the Dirac^1–4^ and Weyl fermions^5–7^. Depending on the inherent symmetry of a particular compound, the crossing points can be several-fold degenerate^8^: two- and four-fold degenerate points are classified as Weyl and Dirac types, respectively. New materials going beyond Weyl and Dirac semimetals with higher band degeneracies have been proposed^8^. Dirac and Weyl semimetals in particular exhibit unusual transport properties, including high mobility^9–11^, large magnetoresistance^9–11^, anomalous Hall effect^12–14^, and a chiral anomaly^14–16^. Although transport phenomena of Weyl and Dirac semimetals have been a topic of intense research, studies on metals with higher degenerate points, for instance, triple point topological metals, are still scarce.

In this article, we have explored the transport properties of the triple point metal MoP, which exhibits an extremely low residual resistivity of 6 nΩcm and very long mean free path of 11 microns despite straightforward synthesis using conventional chemical vapor transport methods. The value of the residual resistivity is on a par with best metals known in the literature. Resistivity is a measure of loss of the charge carrier momentum by large-angle scattering with phonons and impurities. Therefore, large momentum relaxation reflects high resistivity and vice versa. On the other hand, electron–electron and small-angle scattering with phonons conserve momentum within the electron system and, therefore, hardly affect the resistivity^17,18^. The interplay among these scattering processes is at the heart of the hydrodynamic electron flow in materials such as graphene, PdCoO_2_ and WP_2_^19–22^. We show that these scattering processes play a crucial role in determining the excellent transport characteristics of the triple point fermion metal MoP.

## Results

### Crystal structure and electronic structure

**Fig. 1:**
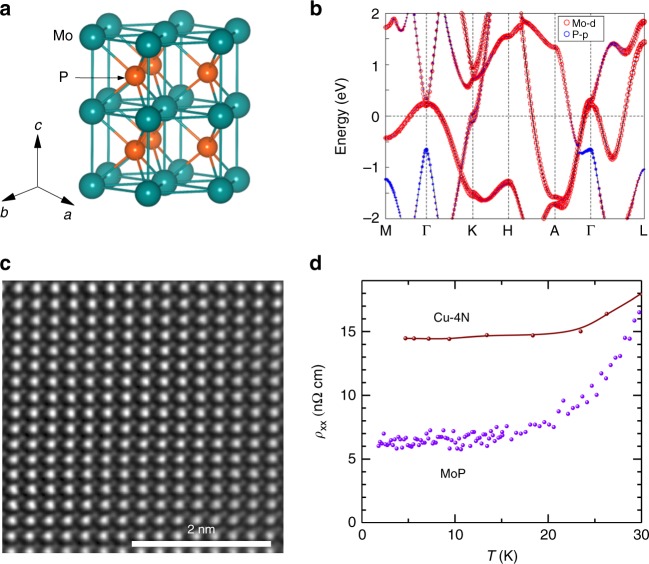
Crystal structure, electronic band structure, high-resolution scanning transmission electron microscopic (HR-STEM) image and resistivity of MoP. **a** Schematic of the hexagonal crystal structure of MoP in which the Mo and P atoms are shown as green and orange spheres, respectively. **b** Energy dispersed band structure along the high symmetry directions, including spin-orbit coupling (SOC). Mo *d*-orbitals contributions (red) dominate at *E*_F_. **c** HR-STEM image along [110], showing Mo (bright large-dots) and P (small-dots). **d** Temperature dependent resistivity, *ρ*_xx_, of MoP compared with 99.99% (4 N) pure Cu^25^

MoP is a simple binary compound, which was first predicted^23^ and then confirmed experimentally to host topologically protected triple point (three-fold degenerate) fermions. It possesses a complex Fermi surface (FS)^24^. Besides the triple point fermions, pairs of Weyl nodes have also been observed experimentally^24^. However, the triple points and Weyl nodes are located far below *E*_F_ (^23,24^ and Supplementary Fig. 1). MoP has a WC-type hexagonal crystal structure (Fig. 1a) belonging to the space group $P\bar 62m$ (No. 187) with lattice parameters *a* = *b* = 3.22 Å and *c* *=* 3.19 Å. Both Mo and P share the same coordination number and coordination environment of six in a trigonal prism. The Mo-P distances are very short and, consequently, the orbitals strongly hybridize with each other and give rise to a complex electronic structure with large bandwidths, as shown in Fig. 1b. Near *E*_F_, the bands are predominantly formed from the *d* orbitals of Mo with minor contributions from the *p* orbitals of P (see Supplementary Fig. 4).

The electrical resistivity, *ρ*_xx_, of MoP crystals, that are grown via chemical vapor transport reactions using standard purity materials (99.999% for P and 99.95% for Mo), reaches an ultra-low value of 6 nΩ cm at *T* = 2 K, which is more than two times lower than Cu of similar purity^25^ (Fig. 1d). The as-grown MoP crystals were analyzed using scanning transmission electron microscopy (high-angle annular dark-field HAADF-STEM). For these STEM studies, ~100 nm thick platelets were prepared by focused ion beam (FIB) milling, with an area of ~5 × ~5 µm^2^. A representative high-resolution micrograph is shown in Fig. 1c, where Mo (bright large-dots) and P (small-dots) atoms are clearly visible. No evidence for defects is found.

**Fig. 2:**
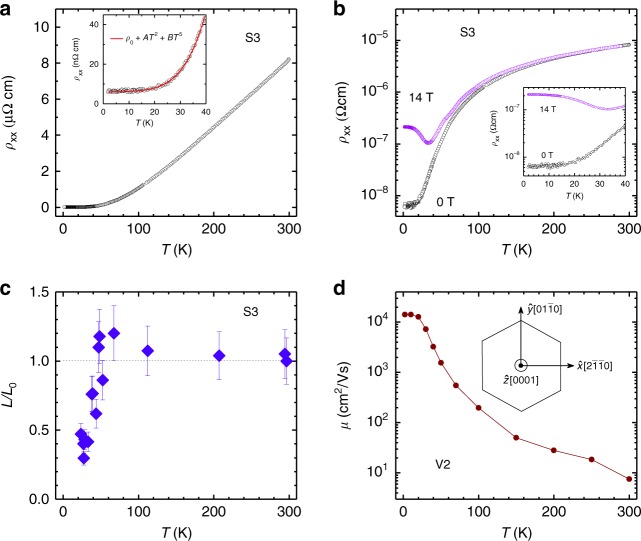
Resistivity, Lorenz number and mobility of MoP. **a** Temperature dependent resistivity, *ρ*_xx_ for ${\mathbf{I}}||{\hat{\mathbf{x}}}$. The inset shows the best fit with *ρ*_xx_ = *ρ*_0_ + *AT*^2^ + *BT*^5^ for the region *T* *<* 40 K, where the values of *ρ*_0_, *A* and *B* are 6.0 × 10^−9^ Ωcm, 2.3 × 10^−12^ ΩcmK^−2^ and 3.6 × 10^−16^ ΩcmK^−5^, respectively. **b** Temperature dependent *ρ*_xx_ at 0 and 14 T when ${\mathbf{I}}||{\hat{\mathbf{x}}}$ and ${\mathbf{B}}||{\hat{\mathbf{z}}}$. The inset shows a significant magnetoresistance below 40 K. **c** Temperature dependent normalized Lorenz number, *L*/*L*_0_, where *L*_0_ is the Lorenz number. The error bar comes from the error in the thermal conductivity measurement (See Supplementary Fig. 7). A strong violation of the Wiedemann–Franz law is observed below 40 K. **d** Temperature dependent charge-carrier mobility, *μ*, of MoP. The inset of **d** shows the crystallographic directions in the hexagonal representation in which ${\hat{\mathbf{x}}}$, ${\hat{\mathbf{y}}}$ and ${\hat{\mathbf{z}}}$ are defined as $[2\bar 1\bar 10]$, $[01\bar 10]$ and [0001], respectively

The morphology of the crystals was analyzed by Laue X-ray diffraction (see Supplementary Fig. 5). The single crystals were then cut into bars whose faces were oriented such that $[2\bar 1\bar 10]$ was along ${\hat{\mathbf{x}}}$, $[01\bar 10]$ was along ${\hat{\mathbf{y}}}$, and [0001] was along ${\hat{\mathbf{z}}}$, as shown in the inset of Fig. 2d. Measurements of the resistivity were made with currents applied along each of these axes for several different crystals (Supplementary Table 1).

### Electrical transport measurements

The temperature dependence of *ρ*_xx_ is shown in Fig. 2a. In zero magnetic field, **B** = 0 (**B** = *μ*_0_**H***)*, no significant difference in *ρ*(*T*) was found for different axes or crystals (Supplementary Fig. 6), notwithstanding the hexagonal symmetry. The material exhibits very low resistivity values at low temperature, which is independent of the crystal orientation. As an example, we consider crystal S3 (Fig. 2a, **I** || ${\hat{\mathbf{x}}}$,). The measured values of *ρ*_xx_ are 6 nΩ cm at 2 K and 8.2 μΩ cm at 300 K, thereby resulting in a very large residual resistivity ratio, RRR = *ρ*_xx_ (300 K)/*ρ*_xx_ (2 K) = 1370. RRR values for other crystals are given in Supplementary Table 1. The RRR is higher than, for example, WC, a triple point fermion semimetal (RRR ~ 85)^26^ and the TaAs family of Weyl semimetals (RRR ~ 115)^11,27^, but lower than, for example, high-quality samples of the Dirac semimetal Cd_3_As_2_ (RRR = 4100)^10^. On the other hand, the residual resistivity, *ρ*_0_ of each of these compounds is much higher than that of MoP (~0.35 μΩcm for WC^26^, ~0.63 μΩcm for NbP^11^ and ~21 nΩcm for Cd_3_As_2_^10^). The temperature dependence of *ρ*_xx_ in **B** = 0 and 14 T (along ${\hat{\mathbf{z}}}$) is compared in Fig. 2b showing that a significant magnetoresistance sets (for other crystals, see supplementary Fig. 9) in below 40 K. This behavior is quite different from conventional metals, such as copper. We fit the low temperature regime of the zero field resistivity according to *ρ*_xx_ $= {\rho} _0 + {AT}^2 + {BT}^5$, where *ρ*_0_ ~ 6.0 × 10^−9^ Ωcm, *A* ~ 2.3 × 10^−12^ ΩcmK^−2^ and *B* ~ 3.6 × 10^−16^ ΩcmK^−5^, respectively. The quadratic term is a ubiquitous feature of Fermi liquids, originating from slow momentum decay via electron–electron scattering. The *T*^5^-term is usually associated with electron–phonon scattering. The value of *A* in MoP exceeds that of *A*_ab_ in PdCoO_2,_ a compound which has shown signatures of a hydrodynamic electron fluid. Importantly, for the free electron gas like aluminum the value of *A* is one order smaller^28^.

### Kadowaki–Woods ratio and Wiedemann–Franz law

To gain further insight into the correlation between charge carriers at low temperatures in MoP, its specific heat *C*_p_ was measured as a function of temperature (Supplementary Fig. 7). The Sommerfeld coefficient, *γ*, and Debye temperature, *Θ*_D_ are 2.37 mJ mol^−1^ K^−2^ and 573 K, respectively. In correlated metals, the pre-factor *A* of the *T*^2^-dependent electrical resistivity and *γ* are fundamentally linked by the modified Kadowaki–Woods ratio (KWR)^29^, because electron–electron interactions enhance both coefficients:

1 $$\frac{{Af_{dx}}}{{{\gamma} ^2}} = \frac{{81}}{{4{\mathrm{\pi }}\hbar k_{\mathrm{B}}^2{\mathrm{e}}^2}} = 1.25 \times 10^{118}\,\Omega {\mathrm{mkg}}^{ - 4}{\mathrm{m}}^{ - 9}{\mathrm{s}}^6{\mathrm{K}}^2,$$

where $f_{dx}$ is a materials specific quantity, *ħ* denotes the reduced Planck constant, $k_{\mathrm{B}}$ is the Boltzmann constant, and e is the elementary charge. We calculate $f_{dx} = \root {3} \of {{3n^7/{\mathrm{\pi }}^4{\hbar} ^6}}$ ^29^. This gives a KWR of $5.1 \ \times 10^{118}$ Ωmkg^−4^m^−9^s^6^K^2^, which is comparable to that calculated for correlated electron systems^29^. In fact, signatures for electron–electron interaction have previously been found in many semimetals such as in Sb^30^, Bi^31^, WP_2_^22^, and PdCoO_2_^21^ at low temperatures. The KWR of MoP highlights once more the importance of the electron–electron interaction in semimetals at low temperatures.

To address the interactions and accompanied scattering mechanism in MoP in more detail, we have next measured the thermal conductivity *κ*, which is related to the electrical conductivity, *σ* through the Wiedemann–Franz (WF) law. According to the WF law *κ*_el_ = *L*_0_*σT*, where *κ*_el_ is the electronic thermal conductivity and *L*_0_ is a universal constant known as the Sommerfeld value of the Lorenz number. The measured temperature dependence of the total thermal conductivity *κ*(*T*) of MoP (crystal S3 with a temperature gradient along ${\hat{\mathbf{x}}}$) increases on decreasing temperature and reaches a value of 3000 WK^−1^m^−1^ at 23 K as shown in Supplementary Fig. 7c. Using the Debye model (see Supplementary Note 6 for details), we extracted *κ*_el_ and calculated the normalized Lorenz number (*L*/*L*_0_) as a function of temperature as shown in Fig. 2c. While the WF law is obeyed at high temperatures, below 40 K, we observe a sudden decrease of *L*/*L*_0_. At 23 K, the value of *L*/*L*_0_ is ~0.4, corresponding to a decrease by 60 %. Such a large deviation from the WF law can also be seen in many conventional metals^32^. The main reason for the deviation from the WF law is the presence of inelastic scattering at intermediate temperatures, which degrade the electrical and thermal currents differently. We will discuss various scattering mechanisms in MoP in the following sections. Interestingly, such a large violation of the WF law was also recently observed in WP_2_ and PtSn_4_; both of them showing evidence towards hydrodynamic fluid^22,33^. However, the correlation between these two observations is yet to be established experimentally.

### Fermi surface topology

To get more quantitative information about the scattering times, we measured the Hall resistivity, *ρ*_yx_, at various temperatures (Supplementary Fig. 8), which can be described by the charge-carrier transport from a single hole band over the entire temperature range from 2 to 300 K. The mobility, *μ*, and carrier density, *n*, obtained from *ρ*_yx_ are 1.4 × 10^4^ cm^2^/Vs, and 3.9 × 10^22^ cm^−3^, respectively, at 2 K, as shown in Fig. 2d and Supplementary Fig. 8d for crystal V2. A small increase in the charge-carrier density with increasing temperature, from 3.9 × 10^22^ cm^−3^ at 2 K, to 1.1 × 10^23^ cm^−3^ at 300 K, is found. However, a much larger decrease in the mobility of ~1000 times is observed over the same temperature range. We studied various MoP crystals grown in two batches (S and V), which are listed in Supplementary Table 1. We find that all the crystals possess a high mobility and large conductivity, *σ*_xx_, as shown in Supplementary Fig. 6b. The values of *σ*_xx_ are mainly controlled by the mobility, which is directly proportional to the momentum-relaxing scattering time, *τ*_mr_, and momentum-relaxing length, *l*_c_, which we determined to be 1.2 × 10^−11^ s and 11 μm at 2 K, respectively, for the crystal V1 (see below for more details). *τ*_mr_ is also called the Drude scattering time, *τ*_mr_ = μm^*^/e. The momentum of the charge carriers is therefore conserved across at least 10^4^ unit cells of MoP.

**Fig. 3:**
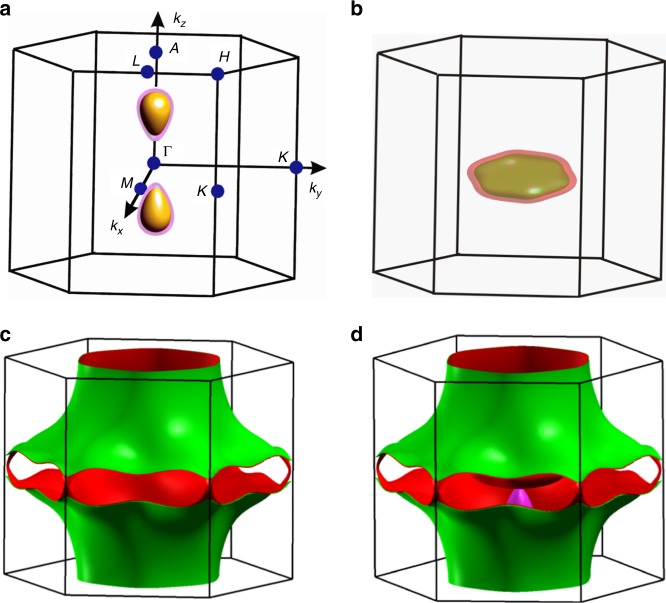
Shape of the Fermi surfaces (FSs) in the first Brillouin zone (BZ) of MoP. **a** Small droplet-type electron pockets which contribute negligibly to the charge-carrier density. **b** Flat hole pockets at the center of the BZ. **c** Open FSs that extend over the entire BZ. **d** Electron pockets, hole pockets, and open FSs combined together in the BZ. Due to the non-centrosymmetric structure of MoP, the pockets as well as the open FSs are spin-split and appear in pairs

To get an estimate of the total amount of scattering events beyond momentum-relaxing processes in MoP at low *T*, we have measured de-Haas van-Alphen (dHvA) oscillations via magnetic torque. This allows for the extraction of the quantum coherence time, which is extremely sensitive to any scattering events that thermalize the electron system. To evaluate these experiments, we have first constructed the FSs with the help of ab-initio electronic structure calculations^34,35^, which are shown in Fig. 3. There are three kinds of FSs; tiny droplet-type electron pockets elongated along the *Γ*-*A* direction (Fig. 3a), disc-shaped hole pockets located around the center of the Brillouin zone (BZ) (Fig. 3b), and typical metallic open FSs extending over almost the whole BZ (Fig. 3c). These FSs are consistent with earlier ARPES experiments^24^. Due to the non-centrosymmetric structure of MoP, all the bands are spin-split^36,37^ resulting in pairs of FSs with similar shapes but different sizes. This spin splitting in a non-magnetic compound (with time reversal symmetry) such as MoP could play a crucial role in the transport properties since it limits back-scattering. We estimate from the band structure calculations that the charge carrier concentration is ∼2.8 × 10^22^ cm^−3^, which is reasonably close to the measured values (2.9-4.3 × 10^22^ cm^−3^ from Supplementary Table 1). These carriers arise largely from the metallic open FSs, with merely ~4% from the closed hole pockets, and a negligible contribution from the electron pockets (<1%). The Fermi velocity, **v**_F_, and the orbital distribution of different FSs are shown in Supplementary Fig. 3, which reveal that the charges in the open FSs and electron pockets have similar **v**_F_.

**Fig. 4:**
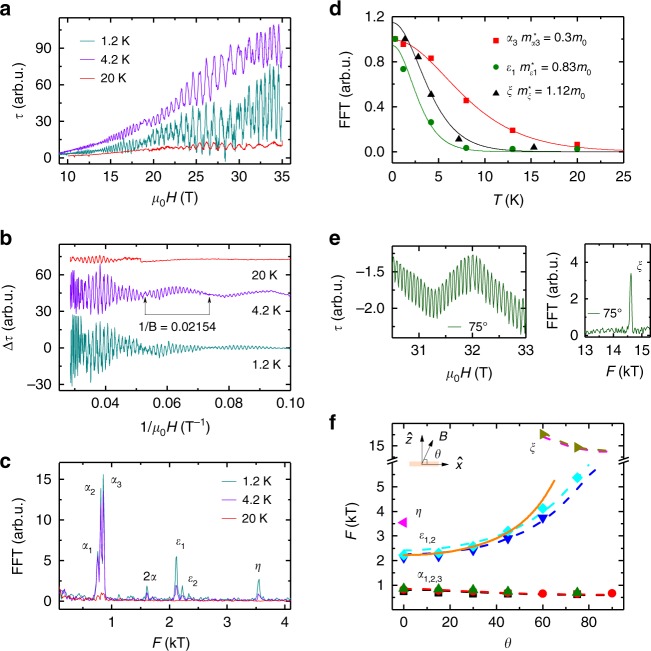
Quantum oscillations, fast Fourier transform (FFT), effective mass, and angular dependent oscillation frequencies of MoP for the crystal V1. **a** de-Haas van-Alphen (dHvA) oscillations from magnetic torque measurements, when ${\mathbf{B}}||{\hat{\mathbf{x}}}$, at various temperatures. **b** Oscillatory components after subtracting a third order polynomial from the data. Beating patterns (marked by arrows) are clearly visible that have a frequency of 45 T, i.e., the difference in frequencies between *α*_2_ and *α*_3_. **c** FFTs from 10–35 T showing different frequencies corresponding to the different pockets involved in the quantum oscillations. **d** Temperature dependence of dHvA amplitudes from FFTs and LK fits giving the effective masses corresponding to different frequencies, *α*_3_, *ε*_1_, and *η*. **e** Magnetic torque signal (*θ* = 75^o^) showing very dense dHvA oscillations: these are visible only when ${\mathbf{B}}$ is nearly along ${\hat{\mathbf{z}}}$ (left panel). The corresponding FFT exhibits a single frequency of 14.6 kT (right panel). **f** Angular dependent frequencies when ${\mathbf{B}}$ is rotated from ${\hat{\mathbf{x}}}$ (*θ* = 0^o^) to ${\hat{\mathbf{z}}}$ (*θ* = 90^o^) where the symbols and dotted lines are experimental and calculated values, respectively. The solid line (orange color) depicts a 1/cos*θ* behavior. The inset shows the rotation geometry of ${\mathbf{B}}$ with respect to the ${\hat{\mathbf{x}}}$ and ${\hat{\mathbf{z}}}$ directions

Having established the fermiology of MoP in theory, torque measurements for ${\mathbf{B}}||{\hat{\mathbf{x}}}$ at various temperatures and the angular dependence in the *xz*-plane at 1.2 K are shown in Fig. 4a and Supplementary Figs 10–12. The dHvA oscillations, which are readily visible up to 20 K are periodic in 1/*B* (Fig. 4b). The frequencies, *F*, of these oscillations are directly related to the extremal cross-sectional areas *A*_F_ of the FS perpendicular to **B** via the Onsager relation $F = \left( {{\it{\Phi }}_0/2{\mathrm{\pi }}^2} \right)A_{\mathrm{F}}$, where ${\it{\Phi }}_0 = h/2{\mathrm{e}}$ (= 2.068 × 10^−15^ Wb) is the magnetic flux quantum and *h* is the Planck constant. Fourier transforms (Fig. 4c, e) of the torque data show fundamental frequencies at *F*_*α*3_ = 855 T, *F*_*ε*__1_ = 2120 T, *F*_*η*_ = 3550 T, and *F*_*ξ*_ = 14,560 T. Such a large frequency of 14,560 T is observed only in some selected materials^21,38,39^. This reveals large Fermi pockets which are usually not expected in typical Dirac^10^ and Weyl semimetals^9,11^. Our observations are in very good agreement with the frequencies derived from our band structure calculations. The dependence of the dHvA frequencies on the orientation (*θ*) of **B** in the *xz*-plane at 1.2 K is shown in Fig. 4f and Supplementary Fig. 11 and displays a 1/cos*θ* behavior at low angles that is expected for a predominantly 2D FS. Since *F*_*η*_ corresponds to the open FSs, these oscillations can only be observed when **B** is close to ${\hat{\mathbf{z}}}$ (*θ* = 90^o^) and, for this reason, we measured the temperature dependence torque signal at *θ* = 75^o^ (Supplementary Fig. 12). Noticeably, the observed oscillations exhibit a clear beating pattern (Fig. 4b) due to the very close frequencies of *α*_2_ (810 T) & *α*_3_ (855 T). These frequencies correspond to spin-split bands due to the lack of inversion symmetry^36,37^. Another spin-split pair of frequencies are *ε*_1_ (2120 T) and *ε*_2_ (2220 T). Effective mass, m^*^, *A*_F_, Fermi wave vector, *k*_F_, and Fermi velocity, **v**_F_, are calculated from the temperature dependence of the oscillation amplitudes (Fig. 4d) using the Lifshitz–Kosevich formula *R*_T_ = *X*/sinh(*X*), where *X* *=* 14.69 m^*^T/*B* and *B* is the average field, and from the Onsager relation, $k_{\mathrm{F}} = \sqrt {A_{\mathrm{F}}/{\mathrm{\pi }}}$, $v_{\mathrm{F}} = k_{\mathrm{F}}h/2{\mathrm{\pi }}m^ {\ast}$, respectively^40^. The resulting values of m^*^, *A*_F_, *k*_F_, *v*_F_ corresponding to the different FS pockets are summarized in Supplementary Table 2. Among the pockets, these values are highest for the open FSs for which we find: m^*^ = 1.12*m*_0_, *A*_F_ = 1.39 Å^−2^, *k*_F_ = 0.67 Å^−1^, *v*_F_ = 6.9 × 10^5^ ms^−1^. The experimental values are consistent with those calculated theoretically. m^*^ for the different bands varies strongly (from 0.23*m*_0_ to 1.12*m*_0_, see Supplementary Table 2), which underlines the complex band structure of MoP. All the bands are spin non-degenerate and the Fermi wave vectors of the spin-split bands *α* and *ε* are separated by 0.004 Å^−1^ (*k*_Fα3_ − *k*_Fα2_ = (0.161 − 0.157) Å^−1^) and 0.006 Å^−1^ (*k*_Fε2_ − *k*_Fε1_ = (0.260 − 0.254) Å^−1^), respectively.

### Momentum relaxing and momentum conserving scattering times

The quantum scattering time is given by ${\tau} _{\mathrm{D}} = \hbar /(2{\mathrm{\pi }}k_{\mathrm{B}}T_{\mathrm{D}})$ or $1.22 \times 10^{ - 12}/T_{\mathrm{D}}$, which we estimate as ${\tau} _{\mathrm{D}}$ = 7.6 × 10^−14^ s at 2 K from the Dingle temperature, *T*_D_ ∼ (1.60 ± 0.15) K. The value of *T*_D_ was estimated from the linear slope of the Dingle plot as shown in Supplementary Fig. 13. The oscillatory behavior of points around the fitted straight line is due to the presence of beating patterns, which are an intrinsic property of the material. At first approximation, the momentum conserving scattering time ${\tau} _{\mathrm{mc}}$ can be estimated by applying Mathiessen’s rule $1/{\tau} _{\mathrm{D}} = 1/{\tau} _{\mathrm{mr}} + 1/{\tau} _{\mathrm{mc}}$_._ Consequently, ${\tau} _{\mathrm{mc}}$ is found to be same order of magnitude as ${\tau} _{\mathrm{D}}$ since $1/{\tau} _{\mathrm{mr}} \, < < \,1/{\tau} _{\mathrm{mc}}$, showing that most of the scatterings in MoP at low temperature conserve the total momentum of the electrons. The value of ${\tau} _{\mathrm{mr}}/{\tau} _{\mathrm{mc}}$ is found to be 15 for MoP.

## Discussion

For hydrodynamic charge flow, the ratio of these scattering times, ${\tau} _{\mathrm{mr}}/{\tau} _{\mathrm{mc}} \, > > \,1$ is the key feature^41^. Similar to the known systems with hydrodynamic charge flow in PdCoO_2_^21^ and WP_2_^22^, this ratio ${\tau} _{\mathrm{mr}}/{\tau} _{\mathrm{mc}}$ in MoP is very high. Interestingly, these compounds have different dimensionalities but they share common properties, such as high conductivity, high magnetoresistance, high mean free path, and electron–electron correlations. MoP, therefore, is a strong candidate to meet the requirements for three-dimensional hydrodynamic charge flow. However, despite the similarities in transport characteristics, an important question remains open and requires careful theoretical and experimental investigation in the future: which microscopic processes make the momentum currents in these materials long lived at low temperatures, even in the limit where the electrical resistivity is limited by impurity scattering?

In summary, MoP is a highly unusual topological metal with high conductivity and mobility, despite no elaborate efforts were taken to eliminate any type of disorder and impurities. The open Fermi surface covers a significant fraction of the volume of the first Brillouin zone and, therefore, is responsible for the high conductivity in MoP. At low temperatures, the compound exhibits an unusually high suppression of momentum relaxation, which is the manifestation of timescales of momentum-relaxing and momentum conserving processes being different by an order of magnitude. MoP is one member of the conventional class of materials that hosts unconventional fermions providing an exciting platform for hydrodynamic electron flow in solids.

## Methods

### Single crystal growth and characterizations

The single crystals of MoP were grown via a simple chemical vapor transport method using iodine as a transport agent. First, MoP polycrystalline powder was synthesized by a direct reaction of molybdenum (Alfa-Aesar, 99.95%) and red phosphorus (Alfa-Aesar, 99.999%) sealed in an evacuated fused silica tube. The sealed tube was heated to 873 K and then to 1073 K. Starting from this powder with iodine, the single crystals were grown in a two-zone furnace at a temperature of 1273 K (T_2_) and 1173 K (T_1_). After several weeks, the ampoule was removed and then quenched in water. Plate-like crystals, of size 0.5–1 mm, were obtained and were further characterized by X-rays and energy dispersive spectroscopy (EDS). The orientation and crystal structure were investigated by Laue X-ray diffraction.

### Electrical and thermal transport measurements

The transport measurements of several MoP single crystals were performed in a physical property measurement systems (PPMS, Quantum Design, ACT option, specific heat option, external low resistivity AC bridge set-up, and customized external thermal transport set-up^42^).

### Magnetic torque measurement

The magnetic torque measurements were performed in an 18 T superconducting magnet at the Dresden High Magnetic Field Laboratory (HLD-EMFL), Helmholtz-Zentrum Dresden-Rossendorf, Germany and up to 35 T in static magnetic fields at the High Field Magnet Laboratory HFML-RU/FOM in Nijmegen, The Netherlands. We paid extra attention to estimate Dingle temperature from quantum oscillations because oscillation amplitude is largely smeared by magnetic field inhomogeneity and limited sampling rate (see Supplementary Fig. 14 and Supplementary Note 8). Besides these, an overlapping field windows with constant width in 1/*μ*_0_*H* for FFT amplitude was selected to minimize the beating effect in Dingle plot. A more detailed discussion can be found in supplementary information.

### Band structure calculations

The electronic structure was calculated by first principles calculations based on density functional theory, using the projected augmented wave method as implemented in the Vienna ab-initio Simulation Package (VASP)^35^. The exchange and correlation energy was considered in the generalized gradient approximation (GGA) with the Perdew-Burke-Ernzerhof-based (PBE) density functional^43^. The tight binding model Hamiltonian was calculated by projected Bloch states onto maximally localized Wannier functions (MLWFs)^44^.

## Supplementary information

Supplementary Information

## Data Availability

The data that support the findings of this study are available from the corresponding author C.S. upon request.
