# Supplementary material for: Extremely high conductivity observed in the triple point topological metal MoP
Source: Nat Commun. 2019 Jun 6;10:2475. doi: 10.1038/s41467-019-10126-y (PMC6554310; doi:10.1038/s41467-019-10126-y)
Supplement: Supplementary file 1 — Supplementary Information [file 41467_2019_10126_MOESM1_ESM.pdf]

SUPPLEMENTARY INFORMATION

Kumar *et al*. Extremely high conductivity observed in the triple point topological metal MoP

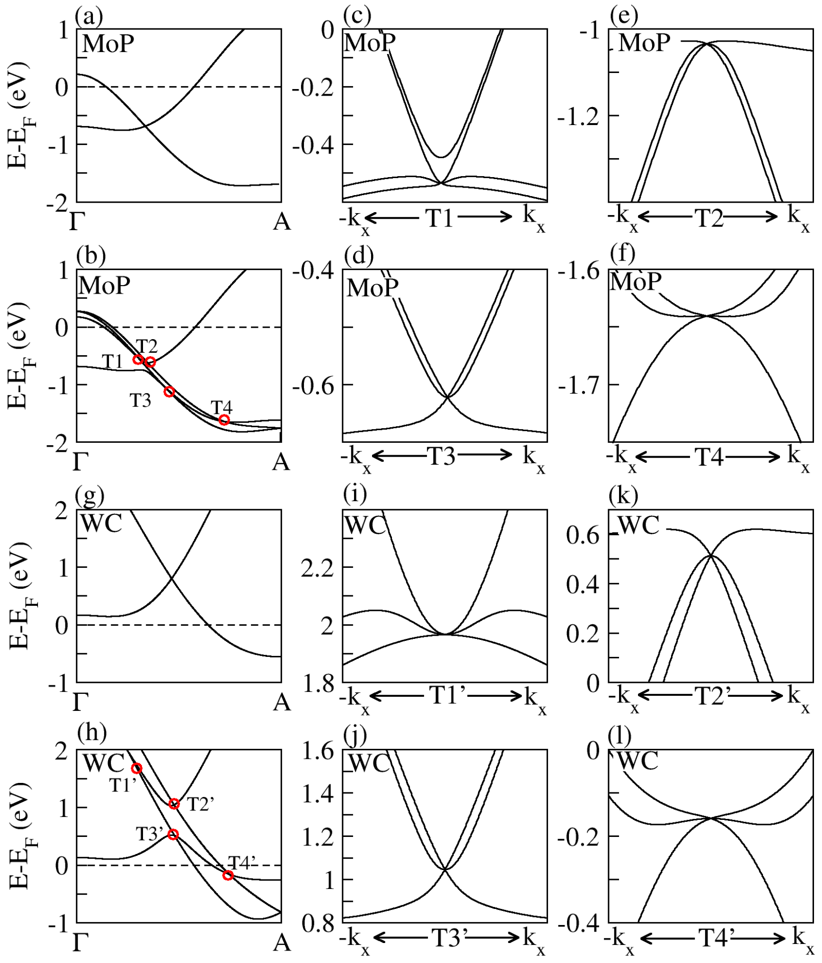

Supplementary Figure 1 | Triple point in MoP and WC. (**a**) and (**b**) Energy dispersion along the high symmetry line Γ-A without and with SOC, respectively. The four triple points are highlighted by the red circles. (**c-f**) Local energy dispersion with crossing of the four triple points along the $\boldsymbol{k_x}$ direction. (**g-l**) Similar plots for WC.

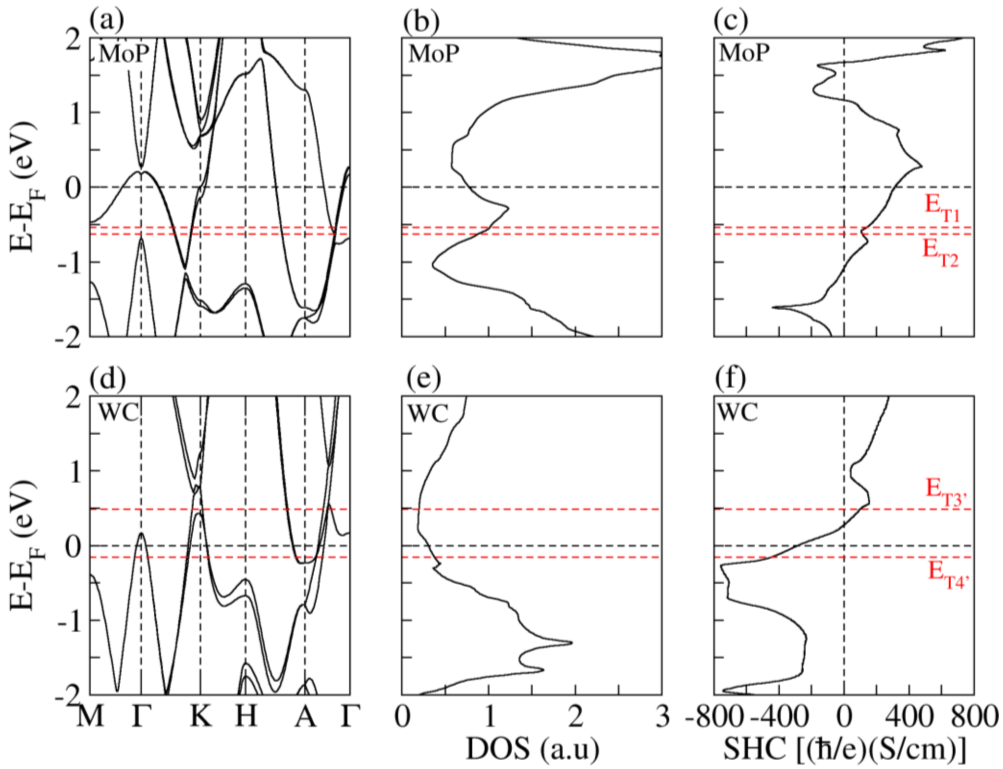

Supplementary Figure 2 | Energy dispersion along high symmetry lines, density of states, and energy dependent spin Hall conductivity (SHC) in (**a-c**) for MoP and (**d-f**) for WC.

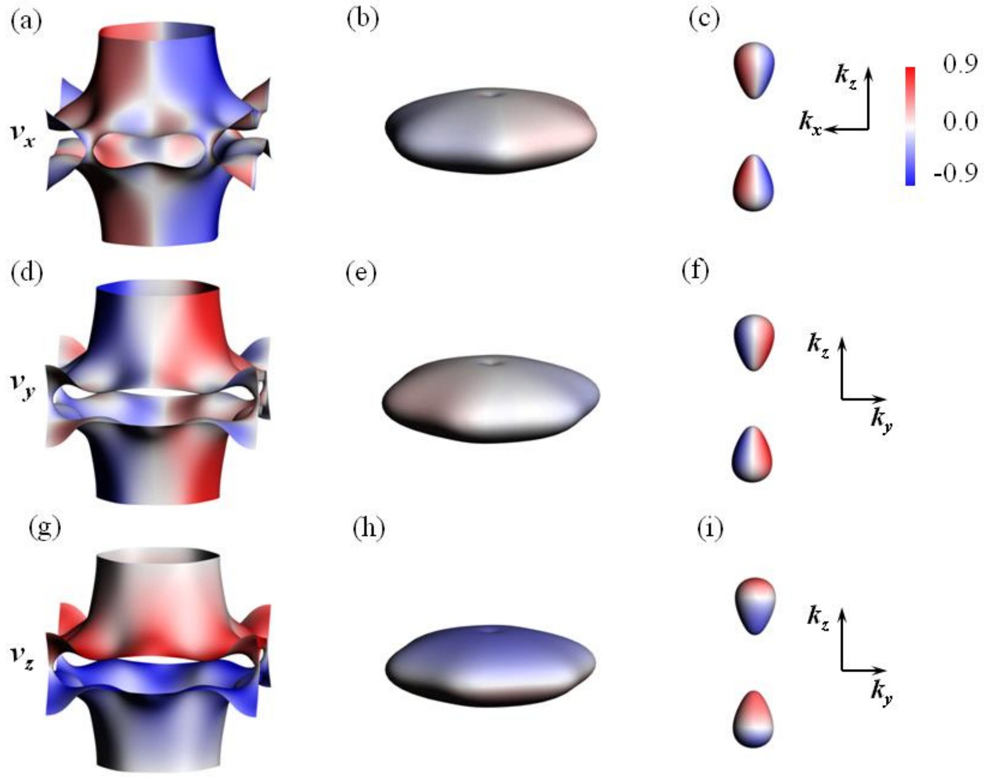

Supplementary Figure 3 | Contributions of the Fermi velocity, $\mathbf{v}_F$, from the charge carriers from different parts of the Fermi surfaces. The three components of (**a-c**) $\mathbf{v}_x$, (**d-f**) $\mathbf{v}_y$, and (**g-i**) $\mathbf{v}_z$ are given separately in the top, middle, and bottom panels, respectively. The maximum Fermi velocity can reach up to 0.9×10^6 m/s. The split Fermi velocity on each pair of FS by SOC is almost the same, we just show one branch for each type of FSs. The color bar is in the unit of 10^6 m/s

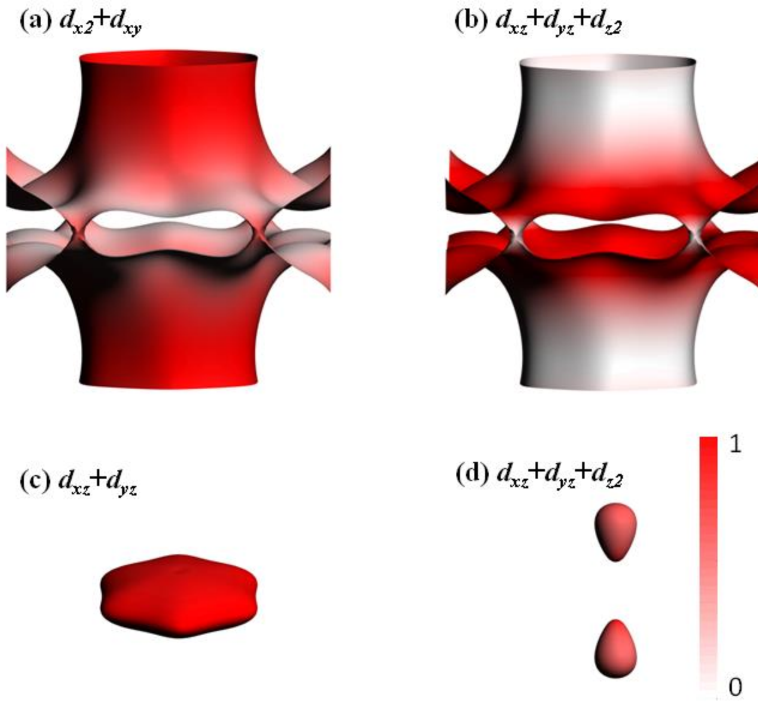

Supplementary Figure 4 | Orbital distribution on the Fermi surface. (**a**) Near the top and bottom of the BZ, the open FSs have mainly from Mo-d_{x2}+d_{xy} character. (**b**) Around the center of the BZ, the open FS are dominated by Mo-d_{xz}+d_{yz}+d_{xz}. (**c**) Hole and (**d**) electron pockets are dominated by Mo-d_{xz}+d_{yz} and Mo-d_{xz}+d_{yz}+d_{z2}, respectively. Spin splitting negligibly affects the orbital distribution of the FSs.

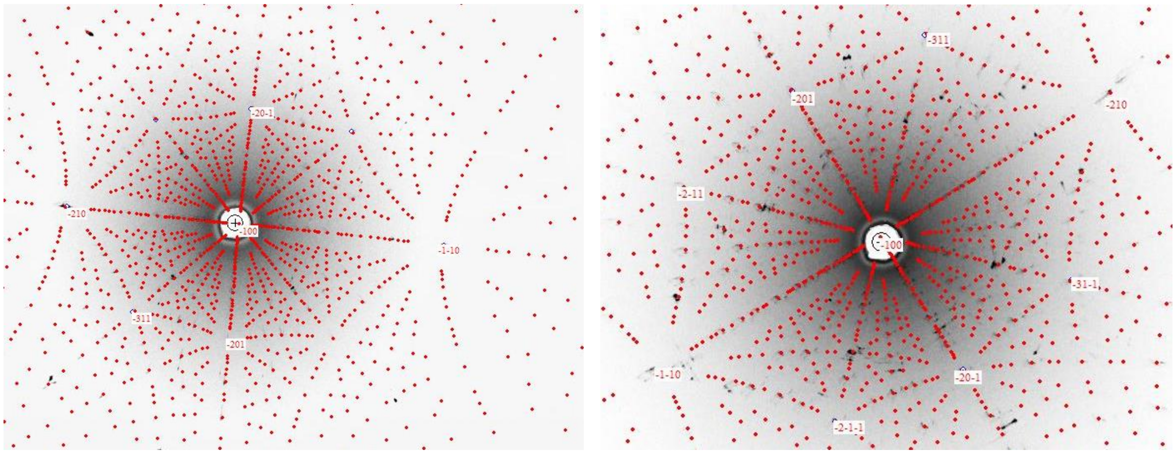

Supplementary Figure 5 | Representative Laue diffraction patterns of as-grown (100) facets of the MoP single crystals S1 (left) and V1 (right). The red spots and assigned Miller indices

show the calculated diffraction pattern of the $P\bar{6}m2$ hexagonal-space group. After identifying the direction, the crystals were cut along desired crystallographic axes for further measurements.

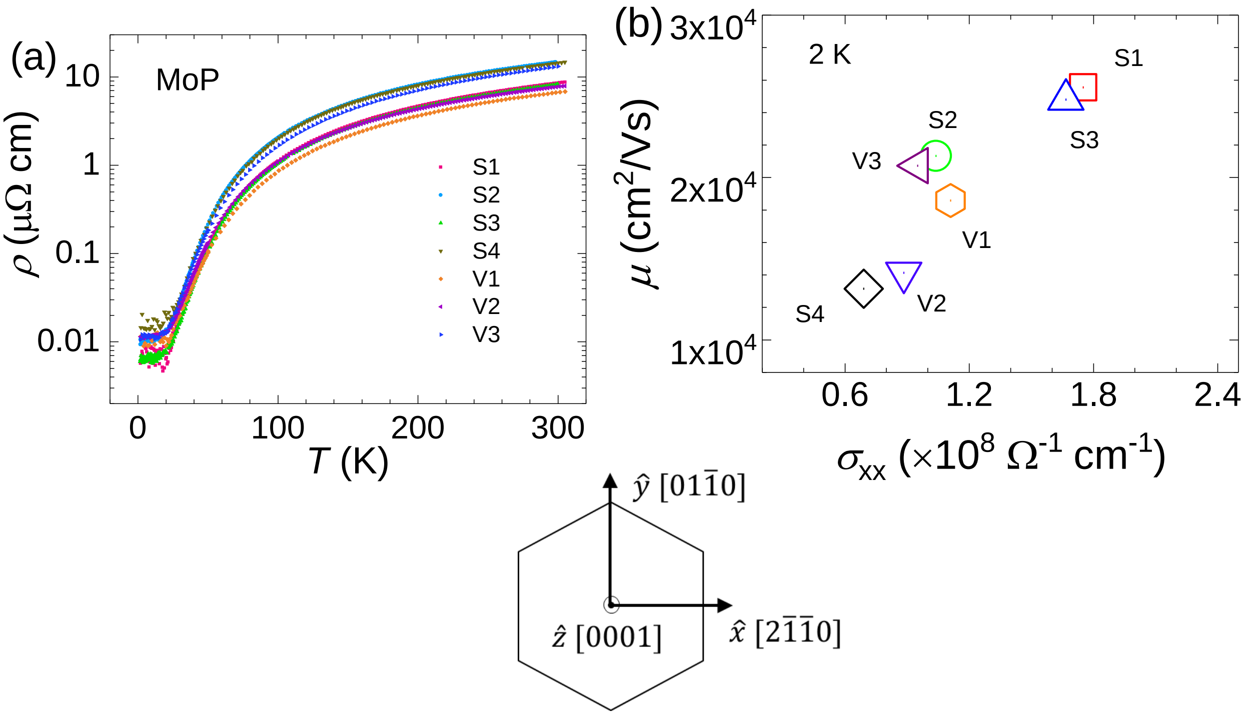

Supplementary Figure 6 | (a) Temperature-dependent resistivity (left panel), ρ, of seven crystals synthesized in two batches (namely S and V). (**b**) Residual conductivity, $\sigma_{xx} = 1/\rho_{xx}$ at $B = 0$, and, mobility, μ of various MoP crystals from two different batches (S,V) at 2 K. The lower panel shows a sketch defining the $\hat{\mathbf{x}}$, $\hat{\mathbf{y}}$, and $\hat{\mathbf{z}}$ directions in terms of hexagonal planes, where $\hat{\mathbf{x}}$ is $2\bar{1}\bar{1}0$, $\hat{\mathbf{y}}$ is $01\bar{1}0$, and $\hat{\mathbf{z}}$ is 0001.

Supplementary Table 1 | Summary of electrical transport parameters of the seven different crystals investigated.

Crystal	Dimension (error: ± 0.005 mm) [length × width × height (l×w×h) mm^3]	Measurement Geometry	ρ at 2K (nΩ cm)	*RRR*	μ at 2K (10^4 cm^{2}V^{-1}s^{-1})	n at 2K (10^{22} cm^{-3})
S1	1.0×0.28×0.20	$\mathbf{I}\|\|\hat{\mathbf{x}}$, $\mathbf{B}\|\|\hat{\mathbf{z}}$	5.7	1526	2.6	4.3
S2	1.0×0.25×0.23	$\mathbf{I}\|\|\hat{\mathbf{z}}$, $\mathbf{B}\|\|\hat{\mathbf{x}}$	9.6	1520	2.1	3.1
S3	0.9×0.23×0.16	$\mathbf{I}\|\|\hat{\mathbf{x}}$, $\mathbf{B}\|\|\hat{\mathbf{z}}$	6	1370	2.5	4.3

S4	1.0×0.1×0.05	$\mathbf{I}\|\|\hat{\mathbf{x}},\ \mathbf{B}\|\|\hat{\mathbf{z}}$	14	1013	1.3	--
V1	0.7×0.22×0.09	$\mathbf{I}\|\|\hat{\mathbf{z}},\ \mathbf{B}\|\|\hat{\mathbf{y}}$	9.2	740	1.9	3.7
V2	1.0×0.25×0.08	$\mathbf{I}\|\|\hat{\mathbf{x}},\ B\|\|\hat{\mathbf{z}}$	11	700	1.4	3.9
V3	0.9×0.15×0.12	$\mathbf{I}\|\|\hat{\mathbf{x}},\ \mathbf{B}\|\|\hat{\mathbf{y}}$	11	1240	2.1	2.9

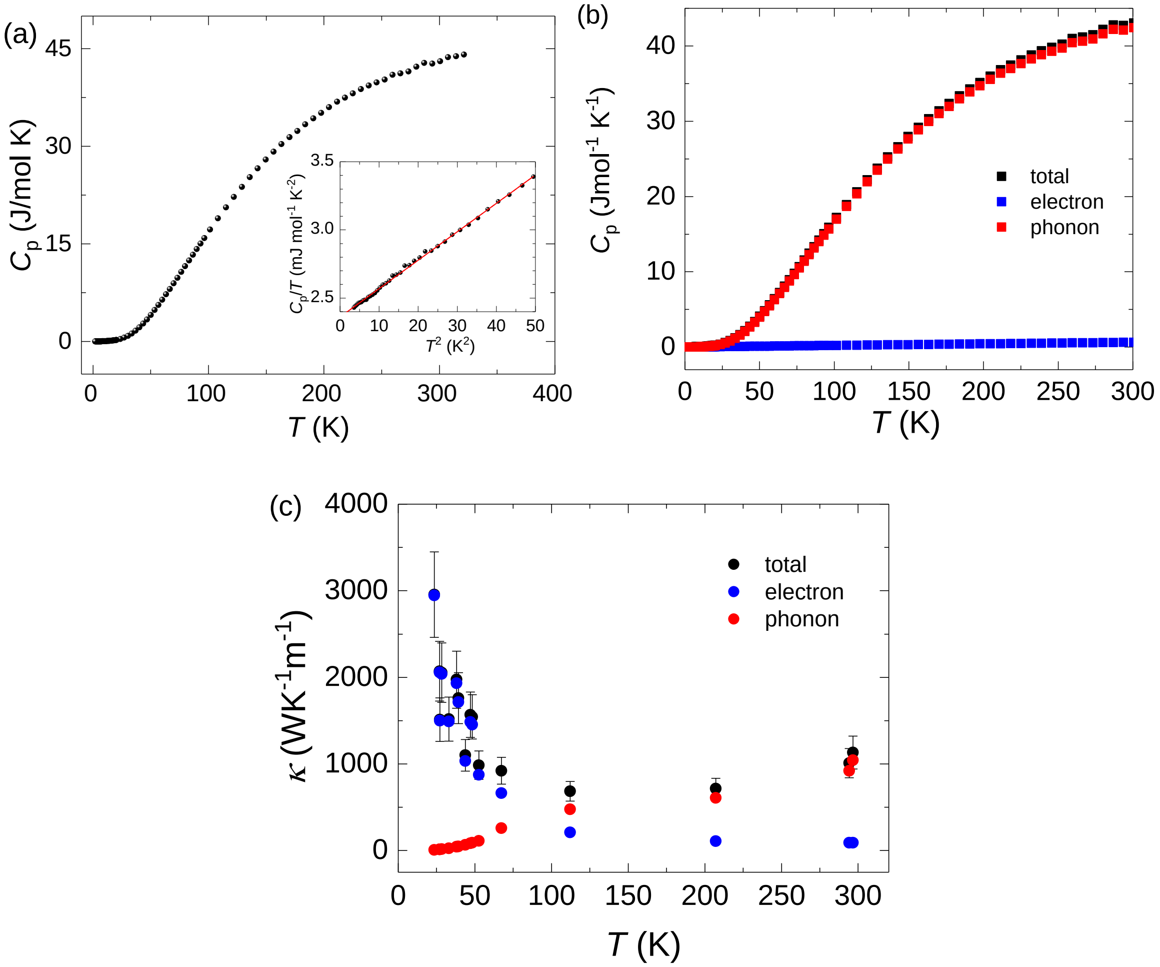

Supplementary Figure 7 | (a) Temperature dependence of the specific heat $C_p(T)$ of MoP. The inset shows a fit (red line) to the data using $C_p(T)/T = \gamma + \beta T^2$. (**b**) Measured temperature dependent total heat capacity, c_p (total), separated phononic part, C_ph, and electronic part, C_el. (**c**) Temperature dependent measured total thermal conductivity, κ (total), calculated phononic (phonon) part from heat capacity and the subtracted electronic part from the measured data. The error bar in the total thermal conductivity data is the standard deviation of systematic error estimated from the uncertainty in thermal gradient and heater voltages.

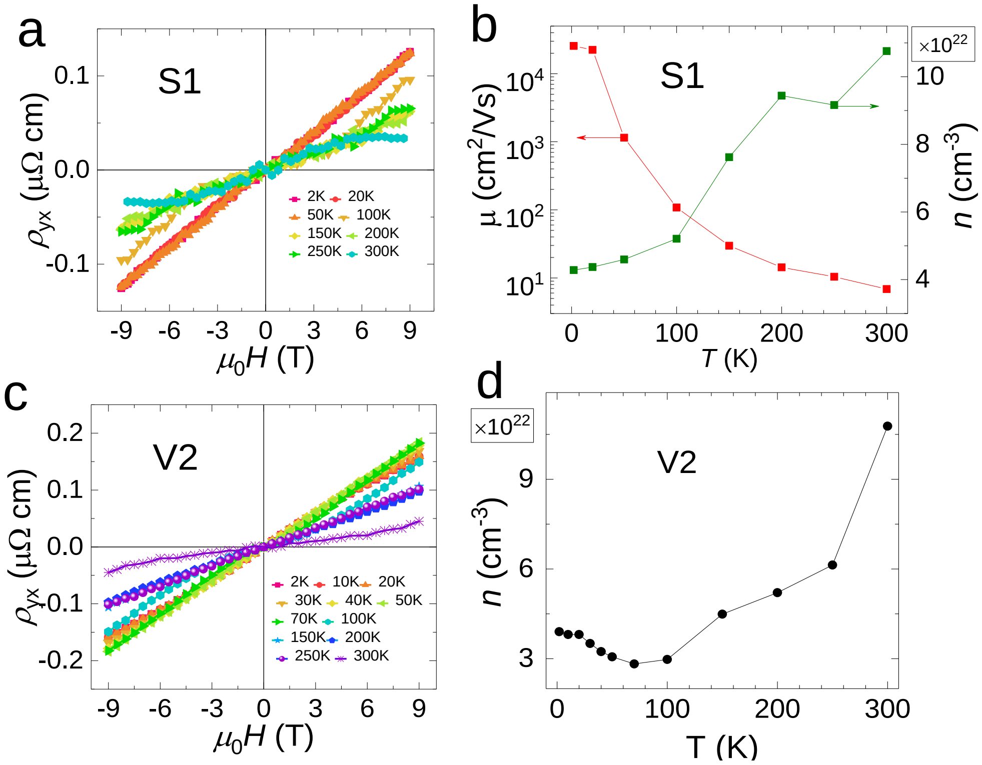

Supplementary Figure 8 | (**a**) and (**c**) Hall resistivity, ρ_{yx}, at various temperatures for crystals S1 and V2. (**b**) and (**d**) estimated temperature dependent mobility, μ, and charge carrier density, n, extracted from the corresponding Hall data using the relations for the Hall coefficient, $R_H = \rho_{yx}/B$, $\mu = R_H/\rho_{xx}$ and $n = 1/eR_H$.

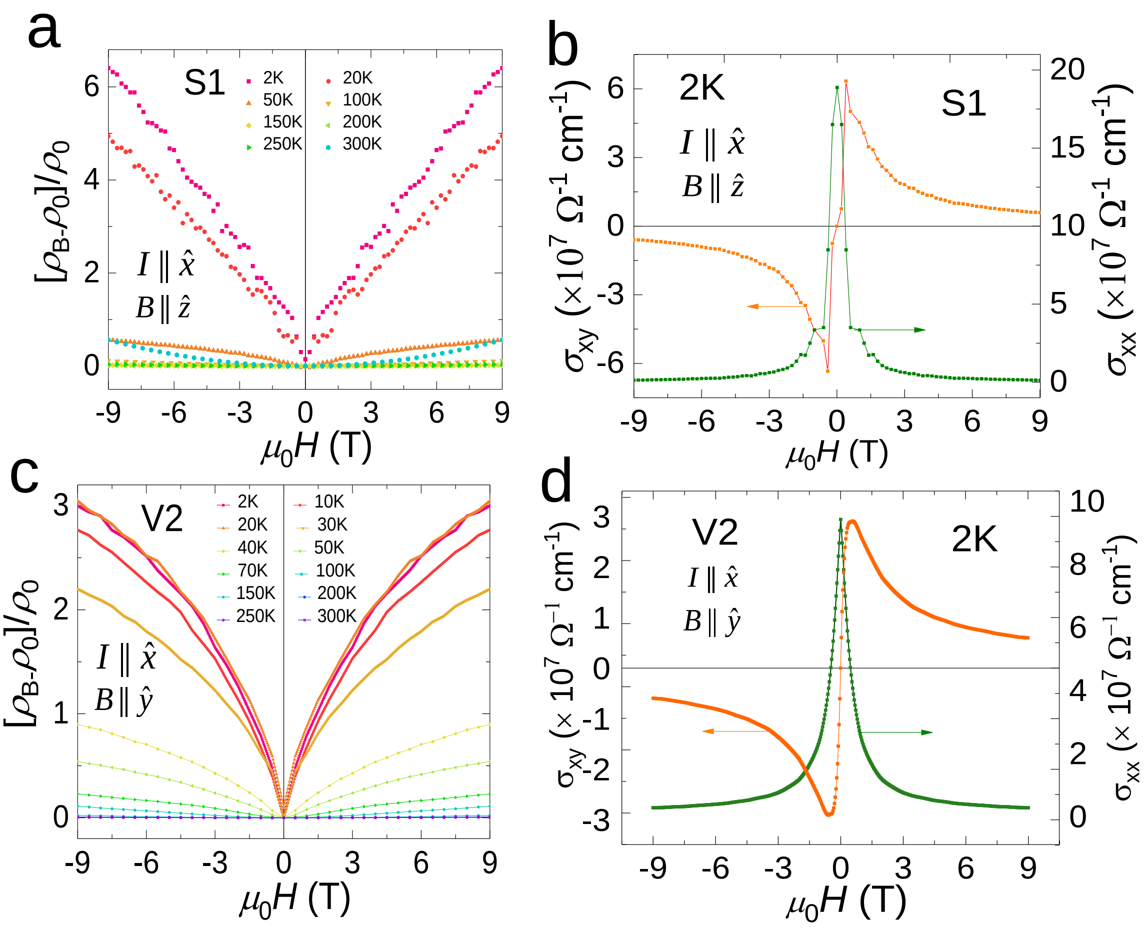

Supplementary Figure 9 | (**a**) and (**c**) Field dependence of the transverse magnetoresistance of crystals S1 and V2. (**b**) and (**d**) corresponding calculated Hall conductivity, σ_{xy}, and transverse conductivity, σ_{xx}, extracted from the relations $\sigma_{xy} = \rho_{yx}/(\rho_{xx}^2 + \rho_{yx}^2)$ and $\sigma_{xx} = \rho_{xx}/(\rho_{xx}^2 + \rho_{yx}^2)$. At low temperature, the magnetoresistance steeply increases at low fields hinting at anti-localization of charge carriers. This effect generally appears in compounds, which possess strong spin-orbit coupling[1,2].

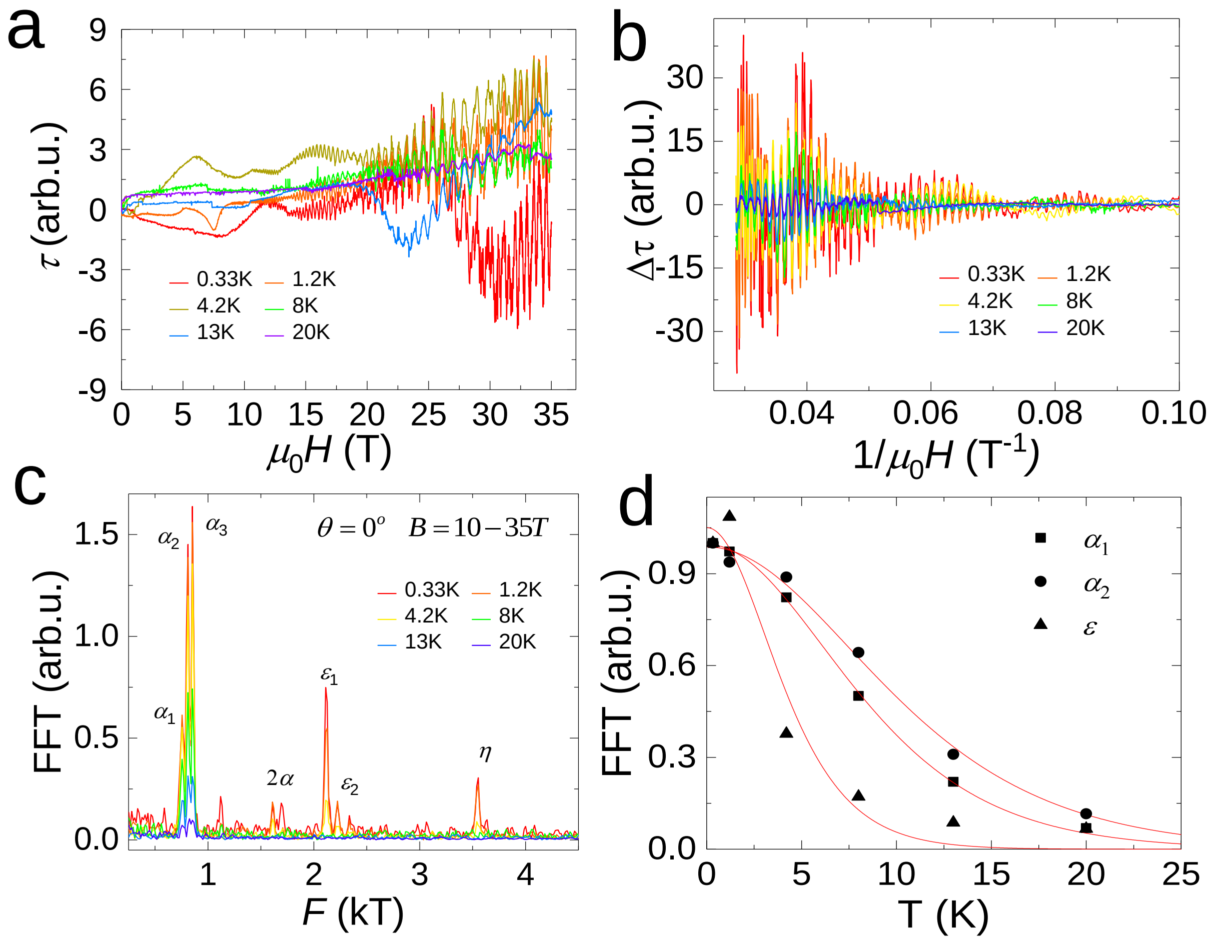

Supplementary Figure 10 | (a) Magnetic torque data at different temperatures. (**b**) dHvA oscillations between 10 and 35 T after subtraction of a smooth background at each temperature. These oscillations are periodic in $1/B$, where $B = 1/\mu_0 H$. (**c**) Fast Fourier transforms (FFTs) of the data shown in (b), which reveals six main frequencies α_1, α_2, α_3, ε_1, ε_2, and η. (**d**) Temperature dependent normalized FFT amplitude of the dHvA signals corresponding to α_1, α_2, and ε_2, with fits using the Lifshitz-Kosevich formula for extracting the effective masses.

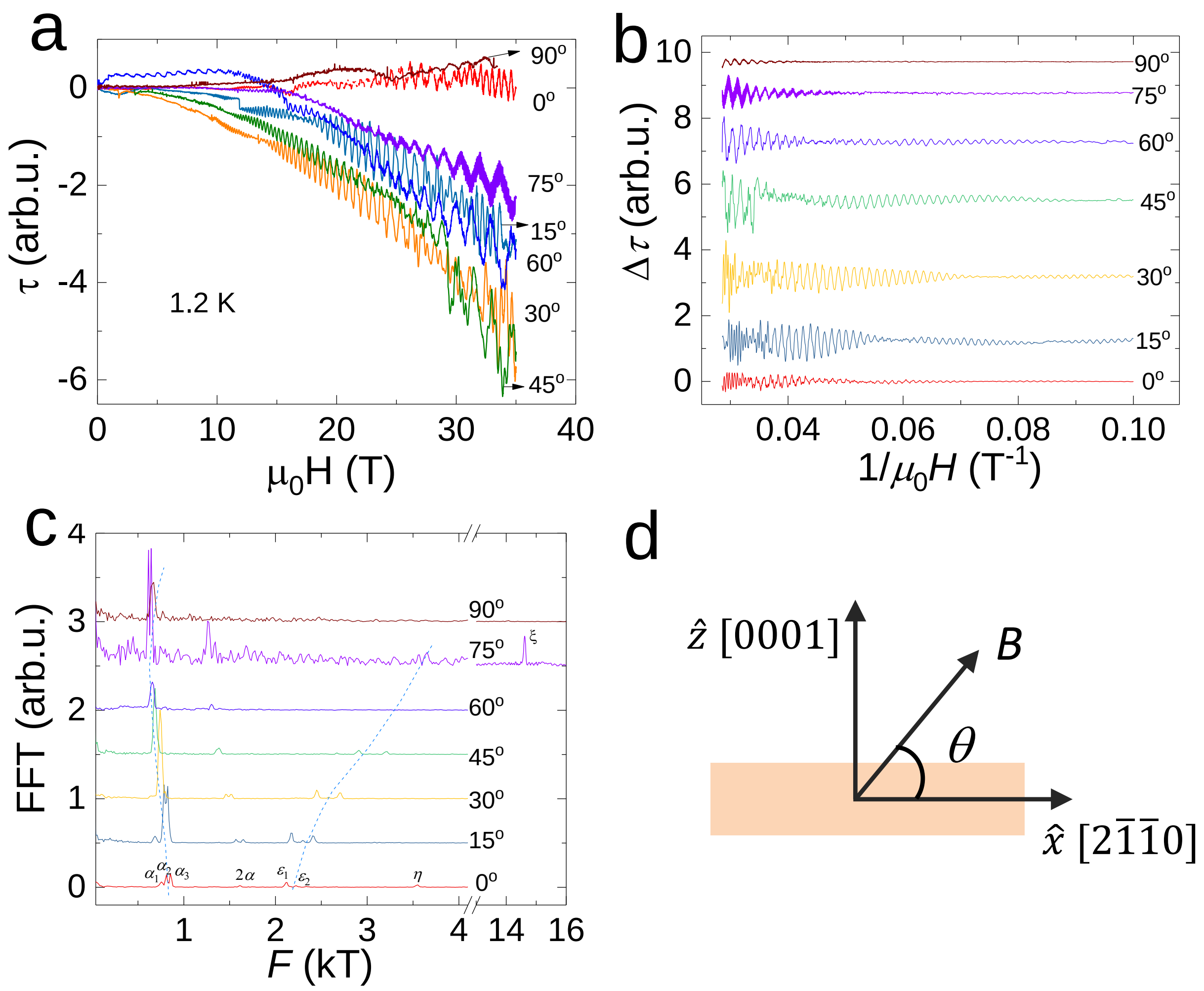

Supplementary Figure 11 | (**a**) Angular dependent magnetic torque data at T = 1.2 K. (**b**) dHvA oscillations between 10 and 35 T after subtraction of a smooth background. (**c**) Fast Fourier transforms (FFTs) of the data shown in **b** in which dotted lines guide the shift of the frequencies with angle. At θ = 70º, a very high frequency appears at 14560 T which is denoted by ξ. (**d**) Schematic of the field rotation direction with respect to the crystallographic direction.

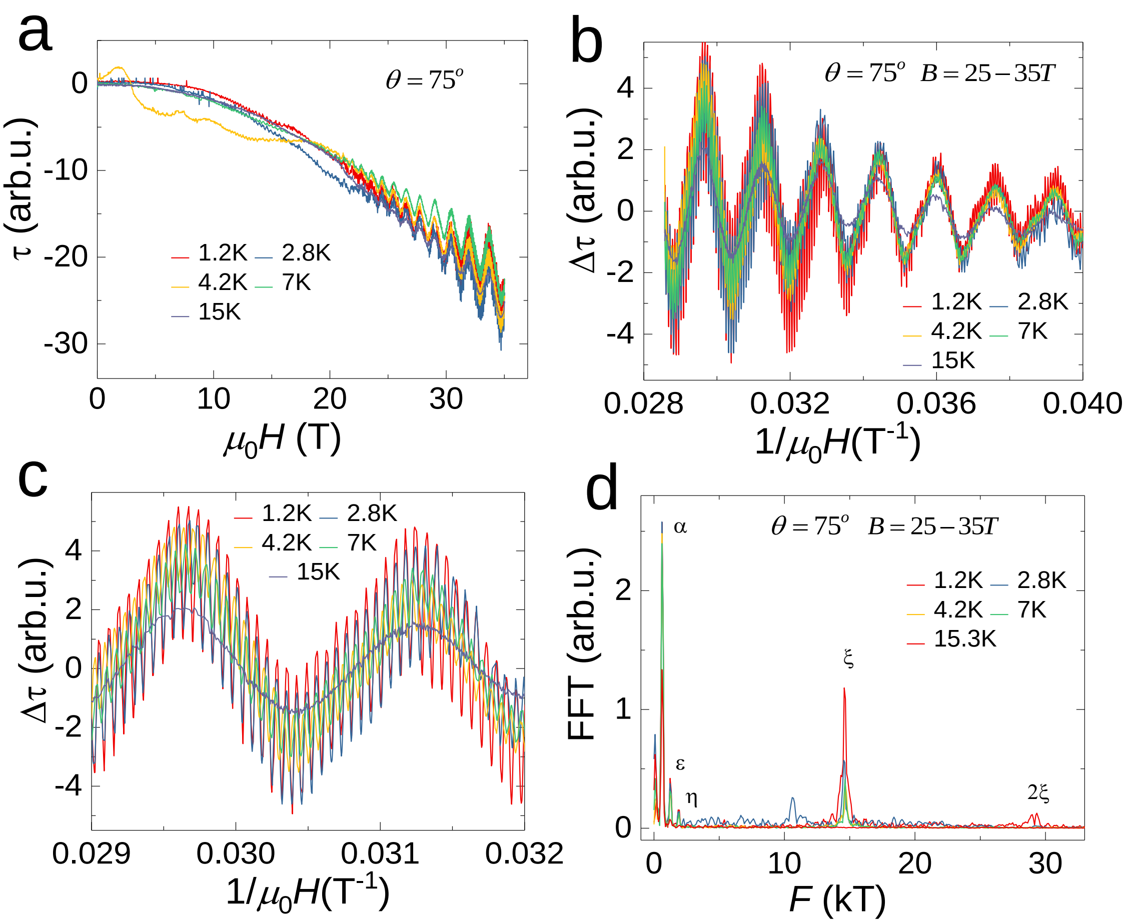

Supplementary Figure 12 | (a) Temperature dependent magnetic torque data at θ = 75°. (**b**) dHvA oscillations between 25 and 35 T after subtraction of a smooth background. (**c**) Zoom of the oscillations shown in (b) in which the high frequency oscillations are clearly visible below 15 K. (**d**) FFT of the data shown in **b**. Besides the frequencies shown in Supplementary Fig. 10, the very high frequency appearing at 14560 T is denoted by ξ.

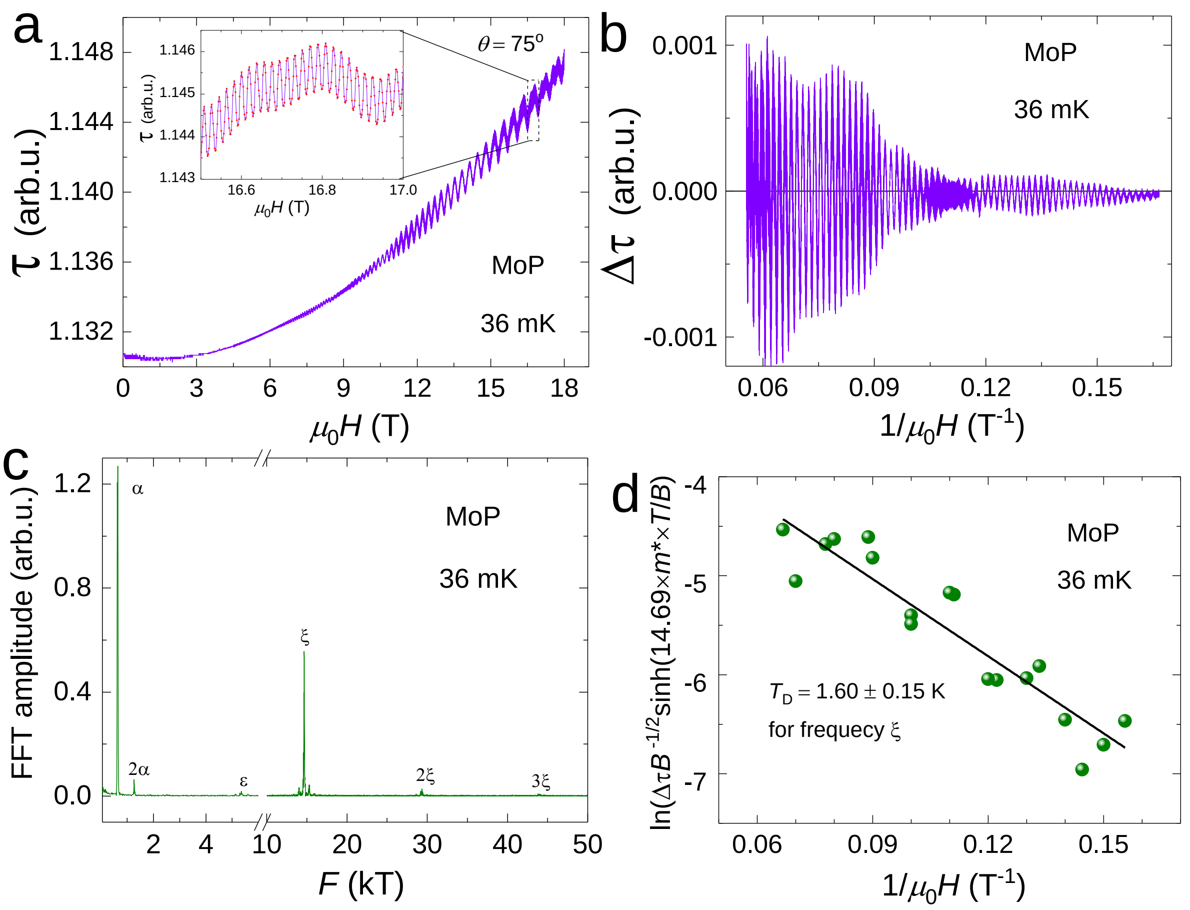

Supplementary Figure 13 | de Haas-van Alphen (dHvA) effect of MoP obtained using an 18 T superconducting magnet at 75° on the same piece of crystal. (**a**) Magnetic torque measurement showing dHvA oscillations at 36 mK. The inset shows an enlargement of the data between 16.5 and 17 T. (**b**) Background subtracted dHvA oscillations between 6 and 18 T. (**c**) Fourier transformation of the data in **b**. Dingle plot of the FFT amplitude of frequency ξ by choosing overlapping field windows with constant width in $1/\mu_0H$.

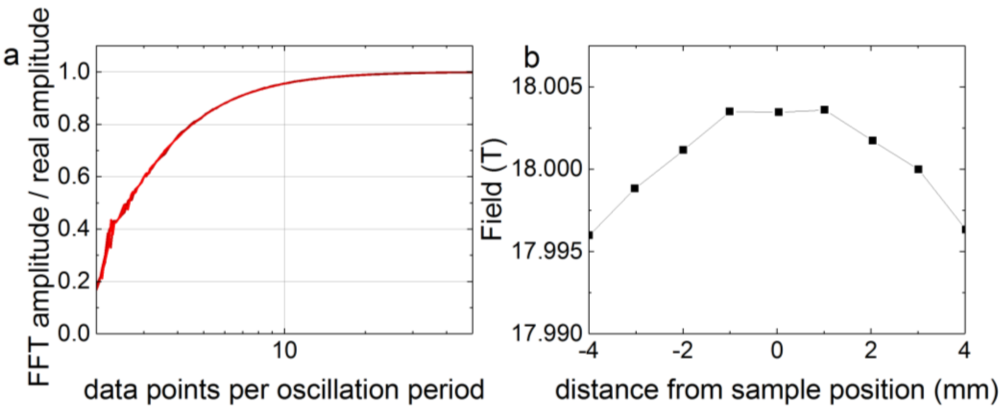

Supplementary Figure 14 | (a) Simulated damping due to limited sampling rate. (**b**) Field profile of the used 18 T superconducting magnet.

Supplementary Table 2 | Oscillation frequency, F, effective mass, m*, extremal enclosed area, A_F, Fermi wave vector, k_F, Fermi velocity, v_F, Drude scattering time, τ_c, and corresponding scattering length, l. These parameters are determined from the magnetic torque oscillations for the crystal V1.

	F (T)	m^*/m_0	A_F (Å^{-2})	k_F (Å^{-1})	v_F (m/s) (×10^5)	τ_c (s) (×10^{-12})	l (μm)
α_1	750	0.23±0.008	0.0715	0.15	7.6	2.4	2.5
α_2	810	0.28±0.004	0.0773	0.157	6.5	3.0	2.6
α_3	855	0.297±0.01	0.0816	0.161	6.4	3.1	2.7
ε_1	2120	0.83±0.09	0.202	0.254	3.5	9.0	4.2
ε_2	2220	0.56±0.027	0.21	0.26	5.4	8.9	4.3
η	3550	0.73±0.05	0.339	0.33	5.2	7.9	5.4
ξ	14560	1.12±0.09	1.39	0.67	6.9	12	11

Supplementary Note 1

Electronic band structure and triple point fermion. The electronic band structure along high symmetry lines of MoP is given in Supplementary Fig. 1b in the main text where we can see that the valence band and conduction bands cross the Fermi energy (E_F). At E_F, the character of Mo d orbitals dominate over the p orbitals of P while they are strongly hybridized. In the absence of spin orbit coupling (SOC), the band inversion between Mo-d_{z2} and -E_g orbitals forms a three-fold degeneracy on the line of Γ-A (Supplementary Fig. 1a), which is protected by C_3 rotation symmetry. Due to the lack of the inversion symmetry in MoP, all such bands are spin non-degenerate at generic k points when including SOC. The bands along Γ-A are reconstructed into two classes by M_z mirror symmetry, two doubly degenerated $|J_z| = 1/2$ bands and two non-degenerated $|J_z| = 3/2$ bands. The crossing between these two types of bands leads to four triple points (TPs) (Supplementary Fig. 1b) which are protected by C_3 symmetry[3,4]. Further, the

dispersions of the four TPs along the k_x direction are shown in Supplementary Fig. 3c-f, where one can see that all these TPs are formed by quadratic bands and further splitting give rise to Weyl points. For comparison, we also analyzed the triple points in WC. Similar to MoP, there are also four triple points along the Γ-A direction. Due to one less valence electron in WC, the three triple points are above the E_F and one below E_F, see Supplementary Fig. 1g-l. As compared to MoP, one triple point is very close to E_F, which is only 0.15 eV below E_F, as shown in the Supplementary Fig. 1k-l.

Supplementary Note 2

Spin Hall conductivity. To check the influence of the triple points, we calculated the spin Hall conductivity (SHC) for MoP and WC, as shown in Supplementary Fig. 2. Remarkably, we noted that the energy dependent peak position of the density of states and SHC (red dotted lines in Supplementary Fig. 2b-c and 2e-f do not appear at the same energy in both MoP and WC. Therefore, the SHC is mainly determined by the band structure. Since the triple points appear normally along with strong entanglement between different bands in an energy window, the peak of the SHC is not lying at the triple points, but it appears near the triple points. Particularly for MoP, the spin Hall conductivity increases from the triple point to the Fermi energy and has a peak slightly above E_F, as shown in Supplementary Fig. 2c. Thus, one can speculate that the presence of topological band may have a nontrivial influence on the transport properties of MoP.

Supplementary Note 3

Fermi velocity and orbital distribution on the Fermi surface. The three components of the Fermi velocity, $\mathbf{v}_x$, $\mathbf{v}_y$, $\mathbf{v}_z$, on the Fermi surface (FS) were calculated by the approximation $v_{f,i} = \frac{\partial E}{\hbar \partial k_i}$, with $i = x$, y, and z. From Supplementary Fig. 3 one can see that the Fermi velocity is locked with the lattice momentum. The maximum Fermi velocity appears on the two open FSs, and parts of them are larger than 0.9×10^6 ms^{-1} that leads to the high mobility in the transport (Supplementary Fig. 3). Around the center of the BZ, the Fermi velocity is mostly dominated by $\mathbf{v}_z$ while $\mathbf{v}_x$+$\mathbf{v}_y$ dominate near the top and bottom of the BZ. The hole pockets centered at the Γ point contribute only to $\mathbf{v}_z$, and the other two components are almost zero. Even though the electron pockets also have a tiny charge carrier concentration, they contribute appreciably to the Fermi velocity. The

open FSs and hole pockets mainly dominate the z components of the Fermi velocity, while the $\mathbf{v}_x$ and $\mathbf{v}_y$ are dominated by the open FSs.

In term of orbitals, the FSs with large v_x and v_y are mainly influenced by the Mo-E_g+d_{xy} orbitals, while $\mathbf{v_z}$ is dominated by the Mo-d_{xz}+d_{yz} orbitals (Supplementary Fig. 4). The relation between the Fermi velocity and orbital distribution of the FSs simply implies that the interlayer hopping of charge carriers between Mo and P determines $\mathbf{v}_z$, while intralayer overlap between Mo controls $\mathbf{v}_x$ and $\mathbf{v}_y$.

Supplementary Note 4

Determination of crystallographic directions. The quality of different MoP crystals and the corresponding orientations were analyzed by white beam backscattering Laue X-ray diffraction at room temperature. The samples show very sharp spots, which can be indexed by a single pattern, suggesting excellent quality of the grown crystals without any twining or domains. Supplementary Fig. 5 represents Laue patterns of crystals superposed with simulated patterns. The data fit with hexagonal $P\bar{6}m2$ crystal structure with lattice parameters $a = b = 3.22$ Å and $c = 3.19$ Å.

Supplementary Note 5

Electrical resistivity. After characterization of two batches of MoP crystals, seven crystals were cut in similar bar shapes but in desired crystallographic directions (see Supplementary Table 1) and four contacts for resistivity and five contacts for Hall resistivity were made using 25 μm Pt wire with Ag paint. Supplementary Table 1 summarizes the crystals' dimension, direction of current, I, magnetic field, B, residual resistivity at T = 2 K, residual resistivity ratio (ρ_{300K}/ρ_{3K}), mobility, μ, and charge carrier density, n, for each crystal. We can see that ρ is independent from the crystallographic direction showing isotropic transport in MoP.

Supplementary Note 6

Specific heat. The specific heat, C_p, was measured on six selected large crystals (Supplementary Fig. 7a). The Dulong-Petit limit $3nR$ (n = number of atoms, R = molar gas constant) is not yet reached at 320 K, indicating relatively strong chemical bonding in MoP. No anomalies from phase transitions are observed in the range covered. The inset shows a C_p/T vs. T^2 representation of the data below T = 7 K. The line indicates a fit of the data to $C_p(T) = \gamma T + \beta T^3$ (inset of the Supplementary Fig. 7), with the conduction electron specific heat γT and the phonon specific heat

in the Debye T^3 approximation. For the (Sommerfeld) coefficient γ, we obtain 2.37 mJ mol^{-1} K^{-2} and β corresponds to a Debye temperature Θ_D = 573 K. The magnetic field dependence of $C_p(T)$ measured at $\mu_0 H$ = 9 T is insignificant. The theoretical integrated density of states (DOS) at E_F is 0.78 states eV^{-1} f.u.$^{-1}$ which is almost equal to the experimentally determined value. A comparison with the measured linear specific heat coefficient, γ = 2.37 mJ mol^{-1} K^{-2}, results in an effective mass ratio m^*/m_e = 1.29. Very similar values of m^*/m_e are observed for simple metals such as Na, K, Rb, Mg, Al and Cu (Ref [5]). Temperature dependent total thermal conductivity, κ (total), calculated phononic (phonon) part using the specific-heat data and remaining electronic part.

The total measured heat capacity, C_{total}(T) = C_{el}(T) + C_{ph}(T), contains an electronic part, C_{el}(T), and a phonon part, C_{ph}(T). At low temperatures (T < 10 K), C_{el}(T) and C_{ph}(T) can be separated by a fit using C_{el}(T) = γT and C_{ph}(T) = βT^3 (T < 10 K).

In contrast to C_{ph}(T) = βT^3, C_{el}(T) = γT is valid over the full temperature range.

Therefore, the phonon contribution is given by

C_{ph}(T) = C_{total} (T) - γT, as shown in Supplementary Fig. 7b.

The total measured thermal conductivity, κ_{total}, is attributed to the electronic part, κ_{el}, and the phononic part, κ_{ph}, i.e.,

$$\kappa_{total}(T) = \kappa_{el}(T) + \kappa_{ph}(T) \qquad (1)$$

Assuming that the Wiedemann-Franz law holds at 300 K, the electronic contribution can be calculated as κ_{el} (300 K) = $L_0\sigma$(300 K) = 89 WK^{-1}m^{-1}, where σ is the measured electrical conductivity at 300 K (1.25 × 10^{7} Ω^{-1} m^{-1}) and L_0 (2.41× 10^{-8} WΩ K^{-2}) is the Lorenz number. Consequently, the phononic contribution of κ at 300 K is:

$$\kappa_{ph}(300\text{ K}) = \kappa_{total}(300\text{ K}) - \kappa_{el}(300\text{ K}) = 1132\text{-}89 = 1043\text{ WK}^{-1}\text{m}^{-1}$$

From the kinetic relation, κ_{ph} (T) is written as:

$$\kappa_{ph}(T) = 1/3\, C_{ph}(T)\, \upsilon^2\, l_{ph}(T), \qquad (2)$$

where υ is the sound velocity and l_{ph} the phonon mean free path. For simplicity, we assume that l_{ph} is independent of temperature. However, l_{ph} is generally increasing with decreasing temperature. By this assumption, we can now calculate the temperature-independent 1/3 υ^2 t_{ph} = κ_{ph} (300 K) / C_{ph}(300 K).

Using $1/3\upsilon^2 t_{ph} C_{ph}(T)$, κ_{ph} (T) can be calculated over the full temperature range and, finally, κ_{el} (T) can be calculated using κ_{el} (T) = κ_{total} (T) - $\kappa_{ph}(T)$. Both, $\kappa_{el\ (T)}$ and κ_{ph} (T) are shown in Supplementary Fig. 7c.

Now, we consider the Lorenz number $L = \kappa_{el}/\sigma T$ in MoP, which helps to characterize thermal and charge transport characteristics. In conventional conductors, the Wiedemann-Franz law holds approximately, i.e., L equals the Lorenz number $L_0 = \pi^2 k_B^2/(3e^2)$. The experimentally extracted κ_{el} as a function of T is given in Supplementary Fig. 7c.

Supplementary Note 7

Magnetic torque. The torque magnetization was measured using capacitive cantilevers mounted on rotatable platforms at the High Field Magnet Laboratory (HFML) in Nijmegen and at the High Magnetic Field Laboratory in Dresden (HLD). The method is based on measuring the change in the capacitance between a reference plate and a flexible CuBe cantilever, on which the sample is attached, as a function of magnetic field. This change in capacitance is due to a slight bending of the cantilever caused by the force exerted by the sample in an external magnetic field. The capacitance was measured using Andeen-Hagerling 2700A capacitance bridges, and the angle with respect to the field was measured using a Hall probe via a Stanford Research SR830 lock-in amplifier (Nijmegen) and via a calibrated stepper motor in Dresden. Initially, the field was applied perpendicular to the cantilever ($B \parallel \hat{\mathbf{x}}$ ($\theta = 0^o$)) and then the sample was rotated stepwise until the sample and cantilever were parallel to the applied field ($B \parallel \hat{\mathbf{z}}$ ($\theta = 90^o$)).

The samples were single crystalline and the perpendicular magnetization component (torque) was measured. The parallel magnetization component (when the sample is placed in a field gradient) was found to be extremely small. Due to the geometry of the experiment, the force from the sample is proportional to Ae_0/C, where C is the measured capacitance, A is defined by the dimensions and force constant of the cantilever, and e_0 is the vacuum permittivity. However, since only relative changes of capacitance (and hence force) are considered in this work, the data

are shown on an arbitrary scale. The magnetic field was determined based on the measured current through the Bitter magnet coils, using calibration curves provided by the HFML.

Supplementary Note 8

Determination of the Dingle temperature. The measured de Haas-van Alphen (dHvA) torque oscillations are well described by the Lifshitz–Kosevich formula [6]

$$\Delta\tau = B^{3/2} R_T R_D \sin\left[2\pi\left(\frac{F}{B}+\gamma\right)\right], \tag{3}$$

where $R_T = \frac{14.69\, m^*\, T/B}{\sinh(14.69\, m^*\, T/B)}$, $R_D = \exp(-14.69\, m^*\, T_D/B)$, in which m^* is the effective mass in units of m_0, T_D is the Dingle temperature, $\frac{2\pi^2 k_B m_0}{\hbar e} = 14.69$ TK^{-1}, m_0 is the bare electron mass, γ is a phase factor, F is the dHvA oscillation frequency, and k_B and ħ are the Boltzmann and reduced Planck constants, respectively. R_D and R_T are related to the broadening of the Landau levels (LL) due to charge-carrier scattering the broadening of the Fermi-Dirac distribution with temperature, respectively. Analysis of the temperature dependent term, R_T, gives the effective mass, whereas the analysis of R_D gives the value of T_D in terms of the scattering time, τ_D, by the relation $\tau_D = \hbar/(2\pi k_B T_D)$. τ_D is known as quantum scattering time which is assumed to be equivalent to the momentum conserving scattering time τ_{mc}.

To calculate T_D, dHvA oscillations were measured via capacitive torque magnetometry at ~36 mK in a superconducting magnet using a ^{3}He/^{4}He dilution refrigerator applying magnetic fields up to 18 T. Initially, the crystal was mounted with $\mathbf{B} \parallel \hat{\mathbf{x}}$ ($\theta = 0^{\circ}$) and dHvA oscillations were measured changing the angle stepwise to track the frequency while rotating towards $\mathbf{B} \parallel \hat{\mathbf{z}}$ ($\theta = 90^{\circ}$). The observed frequencies match closely to those obtained using the 35 T magnet. Since the main interest of this measurement is to calculate T_D for the largest Fermi surface which cuts a nearly circular extremal area normal to the crystallographic *c*-axis of the compound, i.e., *z*-axis, we measured data up to 18 T for an angle of 75° as shown in Supplementary Fig. 13a. The inset of this figure depicts an enlargement of the data between 16.5 and 17 T. The background subtracted dHvA oscillations between 6 to 18 T and their corresponding FFT are shown in Supplementary Fig. 13b and 13c, respectively.

Since the Dingle term varies exponentially with field (Eq. (1)), the so-called Dingle plot of $\ln\left[\Delta\tau \times B^{-1/2} \sinh(14.69\, m^*\, T/B)/\,(14.69\, m^*\, T)\right]/\,14.69\, m^*$ versus $1/B$ follows a straight line where the slope gives the value of T_D. For minimizing the beating effect, we selected

overlapping constant field windows in 1/B for different average fields and determined the amplitude, $\Delta\tau$, from FFTs. The resulting Dingle plot is shown in Supplementary Fig. 13d. The linear slope divided by 14.69m^* yields the value of T_D, which is (1.60 ± 0.15) K for the largest Fermi surface. The oscillatory deviation from the straight line fit is due to the presence of beating caused by the two close spin-split ξ frequencies.

Absence of extrinsic effects in the determined Dingle temperature. Independent of the method used to determine the value of T_D, the oscillations must be free from extrinsic factors such as field inhomogeneity, sweep rate of the magnetic field and magnetic impurities within the sample. From our sample characterizations, we can exclude magnetic impurities in our sample. The magnets used in this measurements are highly precise and homogeneous. The homogeneity of the used 18 T superconducting magnet over the sample is better than 0.2 mT at 18 T, while it is 10^{-4} for the 35 T magnet. It should be noted that the spatial field dependence follows approximately a parabolic function as shown in Supplementary Fig. 14b. In general, the field inhomogeneity, ΔH for a given frequency F should be[6]

$$\Delta H \ll \frac{H^2}{2\pi F} \qquad (4)$$

In order to observe the dHvA frequency of 14600 T in a field of 12 T (average field of our analysis), ΔH should be much smaller than 1.6 mT. The field profile of our 18 T magnet was measured by use of a Hall sensor (Supplementary Fig. 14b). At full field the field inhomogeneity at the sample position, with the sample having dimensions of 1 × 0.7 × 0.2 mm^3, is less than 0.2 mT (that means it is below the resolution of our Hall sensor). Correspondingly, at 12 T the inhomogeneity is less than 0.13 mT. This indeed is much smaller (by more than a factor of 10) than the estimated 1.6 mT.

Another factor which affects the value of T_D is the sampling rate, i.e., the number of data points taken per oscillation period. By adjusting the sweep rate stepwise according to the magnetic field value (0.005 T/min at 4.5 T to 0.1 T/min at 18 T), we ensured the acquisition of at least 12 data points per oscillation period. To estimate the influence of the sweep rate on the dHvA amplitude, the artificial damping due to the limited sampling rate was calculated by generating a sine function of constant amplitude and simulating the loss of amplitude due to decreasing data-point density and averaging effects (Supplementary Fig. 14a). The data measured in the 35 T magnet were taken at a rather fast sweep rate. At the highest field, only 4-5 data

points per period were recorded, which caused an extra damping. Therefore, we refrained from extracting T_D from the data measured in the 35 T magnet.

We conclude that using the data obtained in out 18 T magnet the value of T_D is neither affected by magnetic impurities nor field inhomogeneity nor by a too fast magnetic-field sweep rate.

References

1 Shekhar, C. *et al.* Evidence of surface transport and weak antilocalization in a single crystal of the Bi_2Te_2Se topological insulator. *Phys. Rev. B* **90**, 165140 (2014).

2 Xia, B. *et al.* Indications of surface-dominated transport in single crystalline nanoflake devices of topological insulator $Bi_{1.5}Sb_{0.5}Te_{1.8}Se_{1.2}$. *Phys. Rev. B* **87**, 085442 (2013).

3 Lv, B. Q. *et al.* Observation of three-component fermions in the topological semimetal molybdenum phosphide. *Nature* **546**, 627-631 (2017).

4 Zhu, Z., Winkler, G. W., Wu, Q., Li, J. & Soluyanov, A. A. Triple Point Topological Metals. *Phys. Rev. X* **6**, 031003 (2016).

5 Ashcroft, N. W. & Mermin, N. D. *Solid State Physics (Holt, Rinehart and Winston, New York, 1976)*. 403 (2005).

6 Shoenberg, D. *Magnetic Oscillations in Metals*. (Cambridge Univ. Press, 1984).
